# Population-Based Study Detailing Cutaneous Melanoma Incidence and Mortality Trends in Canada

**DOI:** 10.3389/fmed.2022.830254

**Published:** 2022-03-03

**Authors:** Santina Conte, Feras M. Ghazawi, Michelle Le, Hacene Nedjar, Akram Alakel, François Lagacé, Ilya M. Mukovozov, Janelle Cyr, Ahmed Mourad, Wilson H. Miller, Joël Claveau, Thomas G. Salopek, Elena Netchiporouk, Robert Gniadecki, Denis Sasseville, Elham Rahme, Ivan V. Litvinov

**Affiliations:** ^1^Faculty of Medicine, McGill University, Montréal, QC, Canada; ^2^Division of Dermatology, University of Ottawa, Ottawa, ON, Canada; ^3^Division of Dermatology, McGill University, Montréal, QC, Canada; ^4^Division of Clinical Epidemiology, McGill University, Montréal, QC, Canada; ^5^Department of Dermatology and Skin Science, University of British Columbia, Vancouver, BC, Canada; ^6^Division of Dermatology, University of Toronto, Toronto, ON, Canada; ^7^Division of Dermatology, University of Calgary, Calgary, AB, Canada; ^8^Department of Medicine and Oncology, McGill University Montreal, Montréal, QC, Canada; ^9^Division of Dermatology, Laval University, Quebec City, QC, Canada; ^10^Division of Dermatology, University of Alberta, Edmonton, AB, Canada

**Keywords:** cutaneous melanoma, acral lentiginous melanoma, incidence, mortality, Canada, risk factors, epidemiology

## Abstract

**Background:**

Cutaneous melanoma (CM) is one of the most fatal types of skin cancer. Alarmingly, increases in incidence and mortality were noted globally for this malignancy, despite increase in understanding of melanoma pathogenesis and enhanced prevention efforts.

**Methods:**

Data was extracted for CM patients for provinces and territories (except Quebec) using two independent, population-based registries. Analysis was performed using both clinical and pathological characteristics: tumor morphologic classification, age, sex, anatomic site affected and place of residence. Mortality trends were assessed over a 7-year period. Results were compared to prior findings for 1992–2010.

**Results:**

During 2011–2017 39,610 patients were diagnosed with CM, with 5,890 reported deaths. National crude CM incidence was 20.75 (age-standardized incidence: 14.12) cases per 100,000 individuals per year. Females accounted for 45.8% of cases and 37.1% of deaths. While CM incidence rates continue to increase in both sexes, since 2013 the CM mortality is declining. We observed important differences across the provinces/territories, where Nova Scotia, Prince Edward Island, southern Ontario/British Columbia and certain coastal communities of New Brunswick demonstrated higher CM incidence and mortality rates. The observed incidence and mortality trends for 2011–2017 validate and extend earlier observations from 1992 to 2010 for CM.

**Conclusion:**

This population-based study highlights that while melanoma's incidence is increasing in Canada, mortality rates are for the first time decreasing since 2013. We detail regional distribution of this cancer highlighting communities in southern/coastal areas, as being most at risk as well as the latest trends of melanoma incidence by age, sex and anatomic site. In males, melanoma is more common on the head/trunk, while in females on the extremities. Notably, Acral Lentiginous Melanoma was the only CM subtype that was more common in females, which primarily affects hands and feet.

## Introduction

Cutaneous melanoma (CM) causes more deaths than any other skin cancer (1), accounting for ~1.9 and 1.2% of all cancer deaths in males and females, respectively, in Canada (2). Globally, there were ~290,000 new cases of CM in 2018 (3). Countries with the highest incidence rate per capita include Australia, New Zealand, Norway, Denmark, Netherlands, Sweden and Germany (3). Overall, there was a 44% increase in the incidence rate of CM between 2008 and 2018, with a corresponding surge of 32% in mortality (3). In recent decades, the incidence of CM has been on the rise in fair-skinned individuals in Europe, North America and other parts of the world (4). The incidence rate was further linked to the ongoing climate crisis, as depletion in ozone layer was correlated with subsequent increase in CM incidence (3).

The relationship between ultraviolet (UV) radiation exposure and the risk of developing a skin cancer has been well-established for decades. While solar/artificial UV exposure plays a critical role in the development of melanoma, keratinocyte carcinoma and Merkel cell carcinoma, many host/other exposure factors (Fitzpatrick skin phototype, individual's number of melanocytic nevi, personal/family history of melanoma and other skin cancers, previous therapy with psoralen UVA or broad band UV therapy, history of sunburns, residing closer to the equator or at higher elevation, immunosuppression, other genetic factors/mutations) interplay with the environment to determine the ultimate risk for this deadly disease (5, 6). Despite extensive knowledge on the detrimental impact of UV radiation on skin photoaging, skin cancer development and direct/indirect causation of other common cutaneous diseases [e.g., melasma, rosacea (7–9)], many still fail to exercise sun protection and sun avoidance.

Our group has previously studied the epidemiologic trends of CM in Canada (10–12). In the present study we provide an updated detailed analysis of national CM incidence and mortality trends during 2011–2017 period and compare these findings to the 1992–2010 trends (12).

## Materials and Methods

This study was conducted in accordance with the QICSS-RDC-668035 and 13-SSH-MCG-3749-S001 protocols approved by the Social Sciences and Humanities Research Council of Canada (SSHRC) and the Quebec Inter-University Centre for Social Statistics (QICSS), respectively. In addition, in accordance with the institutional policy, this study received an exemption from the McGill University Research Ethics Board review. We examined the data on the incidence and mortality of CM using two distinct population-based cancer databases: the Canadian Cancer Registry (CCR) and Canadian Vital Statistics (CVS) for the period of 2011–2017 using the International Classification of Diseases for Oncology ICD-O-3 and ICD-10 codes for all subtypes of CM (Supplementary Table 1), as previously reported (10–33). Only invasive melanoma was included in the analysis (i.e., melanoma *in-situ* and lentigo maligna cases were not included). We conducted analyses of the complete data on all CM patients across Canada, with the exception of Quebec, between 2011 and 2017. All crude rates are presented per 100,000 individuals per year. Where indicated in this study, 95% confidence intervals were calculated based on the exact Poisson distribution (10). *P*-values were calculated with the Chi-square goodness of fit test and that *p* < 0.05 were considered statistically significant. Incidence and mortality rates were plotted using linear regression models using GraphPad software to assess trends over time (10). Age-standardized incidence (ASIR) and age-standardized mortality (ASMR) rates for Canada were calculated using the WHO 2000–2025 standard population (10, 34), while in Canadian jurisdictions incidence/mortality was standardized based on Canadian national average, as previously described (10, 11, 16). Geographic maps of Canada divided by FSA codes indicating the residence of patients with CM documented by the CCR or CVS databases were generated using geographic information systems software (ArcMap 10.4; Environmental Systems Research Institute, Redlands, Calif) (10, 22, 23).

## Results

### Incidence Trends of CM

Analysis of invasive CM incidence revealed that there were 39,610 cases diagnosed in Canada (excluding Quebec) during the 2011–2017 period (Table 1A). Quebec was excluded since Le Régistre Québécois du Cancer (LRQC) has not released the data past 2010.

**Table 1:** Clinical characteristics of CM patients in Canada during 2011–2017: (A) analysis by sex and melanoma subtype (B) analysis by age, sex and anatomic site for all CM subtypes.

**A**.							
**CMM Subtypes**	**ICD-O-3 code**	# of patients^*^	**% of total**	**Male (%)**	**Female (%)**	**Mean age ±SD**	***P*-value**
Malignant melanoma, NOS	8720	16,480	41.6	54.3	45.7	62.97 **±** 17.42	<0.001
Superficial spreading melanoma	8743	14,375	36.3	51.1	48.9	60.00 **±** 15.46	0.007
Nodular melanoma	8721	4,390	11.1	59.9	40.1	68.03 **±** 15.56	<0.001
Lentigo maligna melanoma	8742	3,055	7.7	60.2	39.8	72.12 **±** 11.99	<0.001
Acral Lentiginous melanoma	8744	510	1.3	43.1	56.9	66.07 **±** 15.10	0.002
Desmoplastic melanoma	8745	390	1.0	60.3	39.7	70.14 **±** 14.13	<0.001
Amelanotic melanoma	8730	85	0.2	52.9	47.1	68.14 **±** 16.16	0.59
Malignant melanoma, regressing	8723	195	0.5	64.1	35.9	64.07 **±** 14.51	<0.001
Malignant melanoma in junctional nevus	8740	35	0.1	57.1	42.9	61.14 **±** 15.38	0.40
Malignant melanoma in a giant nevus	8761	80	0.2	56.3	43.8	51.76 **±** 17.48	0.26
Balloon cell melanoma	8722	<10	0.03	N/A	N/A	67.25 **±** 15.00	-
Mucosal lentiginous melanoma	8746	<5	0.01	N/A	N/A	66.80 **±** 8.38	-
Overall	-	39,610	100	54.2	45.8	63.26 **±** 16.48	<0.001
**B**.							
		**Males**		**Females**		**Both sexes**	
		#^*^	***%***	#^*^	***%***	#^*^	**%**
Age (years)	0–19	35	0.2	70	0.4	105	0.3
	20–39	1,145	5.	2,045	11.3	3,185	8.0
	40–59	5,735	26.7	6,230	34.3	11,970	30.2
	60–79	10,740	50.0	7,105	39.2	17,845	45.1
	≥80	3,820	17.8	2,685	14.8	6,510	16.4
Anatomic site of melanoma	Skin of lip	25	0.1	30	0.2	55	0.1
	Eyelid	65	0.3	65	0.4	130	0.3
	External ear	795	3.7	195	1.1	990	2.5
	Skin of other parts of face	2,210	10.3	1,500	8.3	3,710	9.4
	Skin of scalp and neck	1,985	9.2	655	3.6	2,640	6.7
	Skin of trunk	8,435	39.3	4,060	22.4	12,500	31.6
	Skin of upper limb and shoulder	4,745	22.1	5,290	29.2	10,035	25.3
	Skin of lower limb and hip	2,155	10.0	5,705	31.5	7,865	19.9
	Overlapping lesion of skin	90	0.4	40	0.2	135	0.3
	Skin, NOS	965	4.5	590	3.4	1,555	3.9
	Total	21,470	100	18,130	100	39,615	100.00

* *Rounded to the nearest 5*.

**Figure 1:**
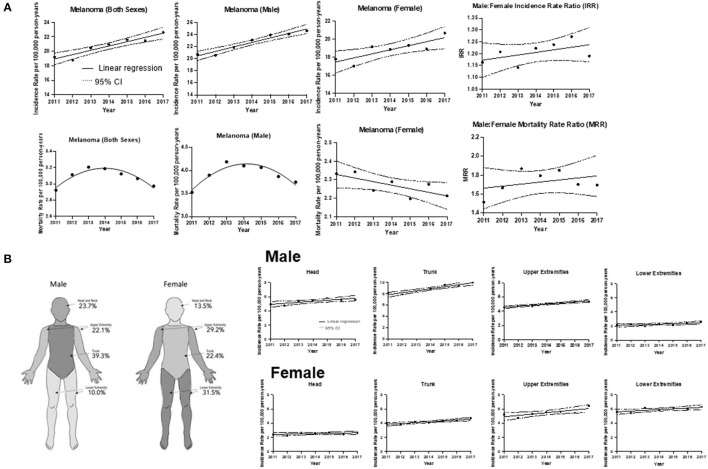
CM incidence and mortality trends. **(A)** Incidence and mortality rates for all cases during 2011–2017 with the line of best fit, and linear regression analysis by sex. The average incidence rate of CM in Canada for both sexes from 2011 to 2017 was 20.75 cases per 100,000 individuals per year. [*R*^2^] = 0.902; *p* = 0.0011. The slope of the line = 0.594 ± 0.0878 cases per 100,000 individuals per year. Incidence of CM for male sex average rate was 22.70. [*R*^2^] = 0.942; *p* = 0.0003. The slope of the line = 0.741 ± 0.0822. Incidence of CM for female sex average rate was 18.84, [*R*^2^] = 0.701; *p* = 0.0188. The slope of the line = 0.449 ± 0.131. Mortality of CM for both sexes (*Y* = −231611 + 230.0X−0.0571X^2^). The average mortality rate in Canada was 3.09 cases per 100,000 individuals per year (95% CI: 3.00–3.16). Mortality of CM for male sex (*Y* = −108970 + 108.2X − 0.0269X^2^). Mortality of CM for female sex, [*R*^2^] = 0.549; *p* = 0.0568. The slope of the line = −0.0193 ± 0.00784. Male to Female Incidence Rate Ratio (IRR). Linear regression analysis of CM incidence rate over time. Coefficient of determination [*R*^2^] = 0.28; *p* = 0.22; CI and the slope of the line = 0.011 ± 0.0078. Line of best fit indicates an average IRR of 1:20:1. Male to Female Mortality Rate Ratio (MRR). [*R*^2^] = 0.14; *p* = 0.4018 and the slope of the line = 0.022 ± 0.024. Line of best fit indicates an average MRR of 1.73:1. **(B)** Trends of CM incidence by anatomic site (head, upper extremities, trunk and lower extremities) over time for males and females. Right panel: Schematic diagram highlighting that the trunk was the most frequent site of CM for male patients, whereas the lower extremities were the most common among female patients. Left panel: Males: head, [*R*^2^] = 0.677, *p* = 0.023 and the slope of the line = 0.14 cases per 100,000 individuals per year; trunk, [*R*^2^] = 0.917; *p* = 0.0007 and the slope of the line = 0.36. For upper extremities, [*R*^2^] = 0.911; *p* = 0.0008 and the slope of the line = 0.146; lower extremities, [*R*^2^] = 0.518; *p* = 0.0682 and the slope of the line = 0.0639. Females: head, [*R*^2^] = 0.288; *p* = 0.2413 and the slope of the line = 0.0393; trunk, [*R*^2^] = 0.817; *p* = 0.0052 and the slope of the line = 0.123; upper extremities, [*R*^2^] = 0.666; *p* = 0.0251 and the slope of the line = 0.192; lower extremities, [*R*^2^] = 0.582; *p* = 0.0460 and the slope of the line = 0.106.

**Figure 2:**
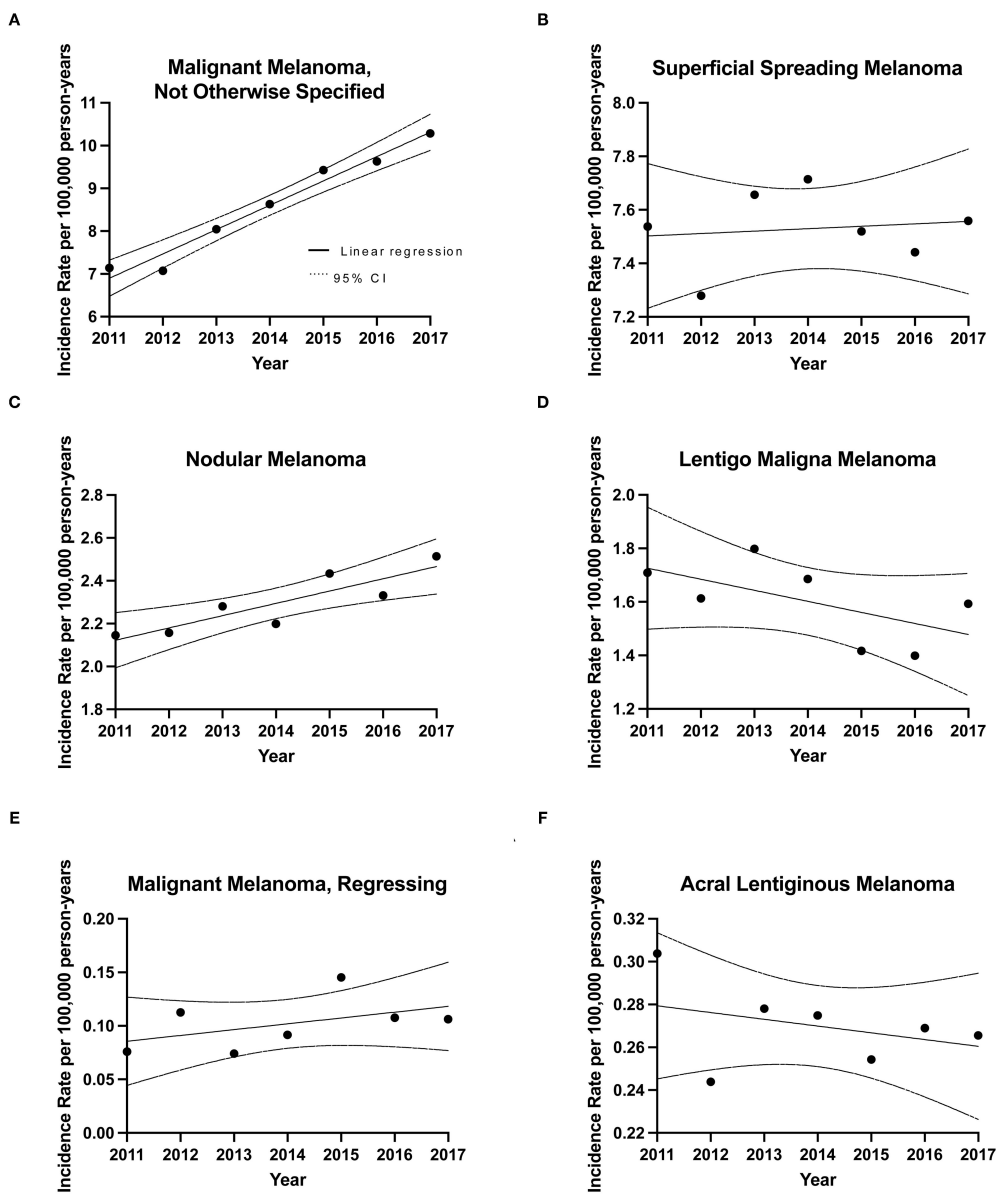
Incidence rates for CM subtypes during 2011–2017. Linear regression analysis of CM subtype incidence rates over time along with CIs. **(A)** Malignant Melanoma, Not Otherwise Specified (NOS) (MM, NOS), slope= 0.569 ± 0.0457, [*R*^2^] = 0.969; *p* < 0.0001, **(B)** Superficial Spreading Melanoma (SSM), slope= 0.00905 ± 0.0292, [*R*^2^] = 0.0188; *p* = 0.7692, **(C)** Nodular melanoma (NM), slope = 0.0574 ± 0.0139, [*R*^2^] = 0.773; *p* = 0.0091, **(D)** lentigo maligna melanoma (LM), slope = −0.0413 ± 0.0246, [*R*^2^] = 0.360; *p* = 0.1541, **(E)** Acral lentiginous melanoma (ALM), slope = −0.00315 ± 0.00369, [*R*^2^] = 0.128; *p* = 0.4312, **(F)** Malignant Melanoma, Regressing (MMR), slope = 0.00543 ± 0.00445, [*R*^2^] = 0.229; *p* = 0.2768.

The average crude incidence rate for CM during 2011–2017 was 20.75 cases per 100,000 individuals per year (95% CI: 20.54–20.95). When compared to the world population (WHO 2000–2025 standard population), the ASIR was 14.12 cases per 100,000 individuals per year (95% CI: 14.10–14.14). For comparison, the 1992–2010 Canadian crude incidence rate was 12.29 and ASIR was 9.63 cases per 100,000 individuals per year. Linear regression analysis of annual incidence highlighted an increasing trend in invasive CM rates across the country, with an annual increase of 0.59 cases per 100,000 individuals (*R*^2^ = 0.90; *p* = 0.0011) (Figure 1). With regards to CM subtypes, the rates of incidence for skin malignant melanoma (not otherwise specified), nodular melanoma, malignant melanoma (regressing) and superficial spreading melanoma increased in 2011–2017, when compared to 1992–2010. In contrast, incidence rates for lentigo maligna melanoma and acral lentiginous melanoma decreased in recent years (Figure 2) (10).

Incidence of other CM subtypes is detailed in Table 1A and Figure 2. Notably, the only subtype that was more common in females was the acral lentiginous melanoma (56.9 vs. 43.1% of cases; *p* = 0.002), which primarily affects hands and feet. The same trend was observed in our 1992–2010 analysis (10).

### Analysis of CM Incidence by Age and Sex

The mean age at diagnosis increased from 58.5 ± 21.6 years during 1992–2010 (10) to 63.3 ± 16.5 years in 2011–2017 years [65.3 ± 14.6 years for males, 60.8 ± 18.2 years for females (Tables 1A,B) provide data for 2011–2017 years, prior data is presented in (10)]. Notably, there was an increase in the number of patients diagnosed with CM >60 years of age, increasing from 48.7% in 1992–2010 to 61.5% in 2011–2017 (Table 1B), compared to 54.0% of males and 42.9% of females being >60, when receiving a diagnosis of melanoma during 1992–2010 (10). The majority of CM subtypes were diagnosed in the 60–79 years of age bracket (Tables 1A,B). Based on 1992–2010 data, most subtypes were diagnosed in their late 50's and early 60's with the exception of patients with desmoplastic melanoma and lentigo maligna melanoma, where average age at diagnosis was 70.1 and 72.1 years, respectively. The diagnosis age for these two subtypes remained unchanged for 1992–2010 and 2011–2017 years (10).

With regards to the analysis by sex, we continued to observe a higher incidence in males than in females (54.2 *vs*. 45.8%), and this trend remained consistent throughout the majority of CM subtypes (Table 1A). Figure 1A depicts the male-to-female incidence rate ratios (IRR) for 2011 to 2017. The mean IRR for this period was 1.20, signifying an ever-increasing male to female ratio. This is consistent with previously noted trends (IRR of 1.12 for 1992–2010) (10). While the overall incidence was higher in males than in females, young (0–39 years) and middle-aged (40–59) females had a higher incidence rate than their male counterparts.

### Analysis of CM by Anatomic Site

Analysis of CM by anatomic location revealed similar trends to those observed in 1992–2010 (10). The majority (63.0%) of CM in males developed on the head, neck and trunk, while in females, upper and lower extremities accounted >60% of cases. Overall, there was an increase in incidence rates in all anatomic sites in both sexes, with the trunk and upper extremities exhibiting the most significant increase in incidence (Figure 1B). The annual rate of increase of CM incidence (using the line of best fit) on the trunk in males was 0.36 cases per 100,000 individuals, while it was 0.19 cases per 100,000 individuals for the upper extremities of females (Figure 1B). We observed a substantial increase in incidence of CM on the trunk in males (0.14 cases per 100,000 individuals per year during 1992–2010 and 0.36 during 2011–2017) on the trunk in females (0.062 during 1992–2010 and 0.123 during 2011–2017) and on upper extremities in females (0.086 during 1992–2010 and 0.192 during 2011–2017).

### Geographic Distribution of CM

When analyzing CM rates by province, we discovered that maritime provinces of Prince Edward Island (crude incidence rate of 33.86 cases per 100,000 individuals per year), Nova Scotia (30.77) had notably high incidence rates [Tables 2A,B; crude and age-standardized rates (ASIR) provided]. CM crude incidence rates in New Brunswick (22.55), Ontario (22.47) and British Columbia (20.89) were higher, but comparable to the national average of 20.75 cases per 100,000 individuals per year. Newfoundland and Labrador, territories and the prairie provinces had lower rates than the Canadian average. Adjusting for age further confirmed these findings (Table 2). Nova Scotia and Prince Edward Island demonstrated the highest incidence rates in the country based on our 1992–2010 results (10). Notably, all provinces demonstrated an appreciable increase in melanoma incidence in recent years.

Table 2Distribution of CM patient (A) incidence and B) mortality in Canadian provinces and territories. Incidence rate is per 100,000 individuals per year. (C) Clinical characteristics (anatomic site involved, sex and age) of CM in deceased individuals during 2011–2017.

**Table:** 

**A**.									
**Province**	Cases^*^	**Population^‡^**	**Crude Incidence**	**Lower CI (95%)**	**Upper CI (95%)**	**Age-standardized incidence**	**Variance Rate, Poisson**	**Lower CI (95%)**	**Upper CI (95%)**
Nova Scotia^§^	2,030	942,000	30.77	29.45	32.14	27.66	0.38	26.45	28.87
Prince Edward Island^§^	345	146,000	33.86	30.38	37.63	30.94	2.80	27.66	34.22
British Columbia^±^	11,830	4,709,000	20.89	20.39	21.38	19.78	0.057	19.31	20.25
New Brunswick^§^	1,200	760,000	22.55	21.20	23.77	19.99	0.34	18.85	21.13
Ontario^§^	21,445	13,634,000	22.47	22.17	22.77	22.24	0.023	21.95	22.54
Alberta^#^	4,385	4,045,000	15.49	15.03	15.95	17.91	0.074	17.38	18.44
Saskatchewan^#^	1,125	1,110,000	14.49	13.64	15.35	15.14	0.20	14.25	16.03
Manitoba^#^	1,460	1,281,000	16.28	15.45	17.14	16.99	0.20	16.12	17.86
Newfoundland and Labrador^#^	690	528,000	18.69	17.19	19.99	16.63	0.41	15.37	17.88
Northern Territories^#^	50	117,000	6.09	4.52	9.03	7.93	1.33	5.67	10.19
Canada (excluding Quebec)	39,615	27,272,000	20.75	20.54	20.95	N/A^&^	N/A^&^	N/A^&^	N/A^&^
**B**.									
Nova Scotia^§^	280	942,000	4.24	3.76	4.77	3.80	0.052	3.35	4.25
Prince Edward Island^§^	50	146,000	4.91	3.64	6.47	4.51	0.411	3.26	5.77
British Columbia^#^	940	4,709,000	2.85	2.67	3.04	2.69	0.0077	2.52	2.87
New Brunswick^±^	165	760,000	3.10	2.65	3.61	2.76	0.047	2.33	3.18
Ontario^§^	3,360	13,634,000	3.52	3.40	3.64	3.48	0.0036	3.36	3.60
Alberta^#^	585	4,045,000	2.07	1.90	2.24	2.46	0.011	2.26	2.66
Saskatchewan^#^	190	1,110,000	2.45	2.11	2.82	2.48	0.033	2.13	2.83
Manitoba^#^	215	1,281,000	2.40	2.09	2.74	2.43	0.028	2.11	2.76
Newfoundland and Labrador^#^	95	528,000	2.57	2.08	3.15	2.38	0.061	1.89	2.86
Canada (excluding Quebec)	5,880	27,272,000	3.08	3.00	3.16	N/A^&^	N/A^&^	N/A^&^	N/A^&^

**Table:** 

**C**.									
**CM Mortality Trends**		Number of patients^*^	**% of reported case**
Anatomical site	Lip + eyelid, including canthus	N/A	N/A
	Ear and external auricular canal	30	0.5
	Other and unspecified parts of face	90	1.5
	Scalp and neck	95	1.6
	Trunk	230	3.9
	Upper limb, including shoulder	115	2.0
	Lower limb, including hip	165	2.8
	Skin, unspecified	5,155	87.7
	Total	5,880	100
Sex	Male^*^	3,705	62.9
	Female^*^	2,185	37.1
Age (Both sexes)	0–19	N/A	N/A
	20–39	210	3.6
	40–59	1,240	21.1
	60–79	2,715	46.1
	80+	1,720	29.2
Age (Males)	0–19	N/A	N/A
	20–39	120	3.2
	40–59	755	20.4
	60–79	1,805	48.7
	80+	1,030	27.8
Age (Females)	0–19	N/A	N/A
	20–39	100	4.6
	40–59	480	22.1
	60–79	905	41.6
	80+	690	31.7

*Case counts are rounded to the nearest 5*.

‡ *All population numbers are rounded to the nearest thousand*.

* *p <0.01*.

§ *Statistically significant higher rates than the national average*.

# *Statistically significant lower rates than the national average*.

± *Statistically not significant rates*.

& *The rates are adjusted to the Canadian average and, hence, the national rates remain unchanged*.

**Figure 3:**
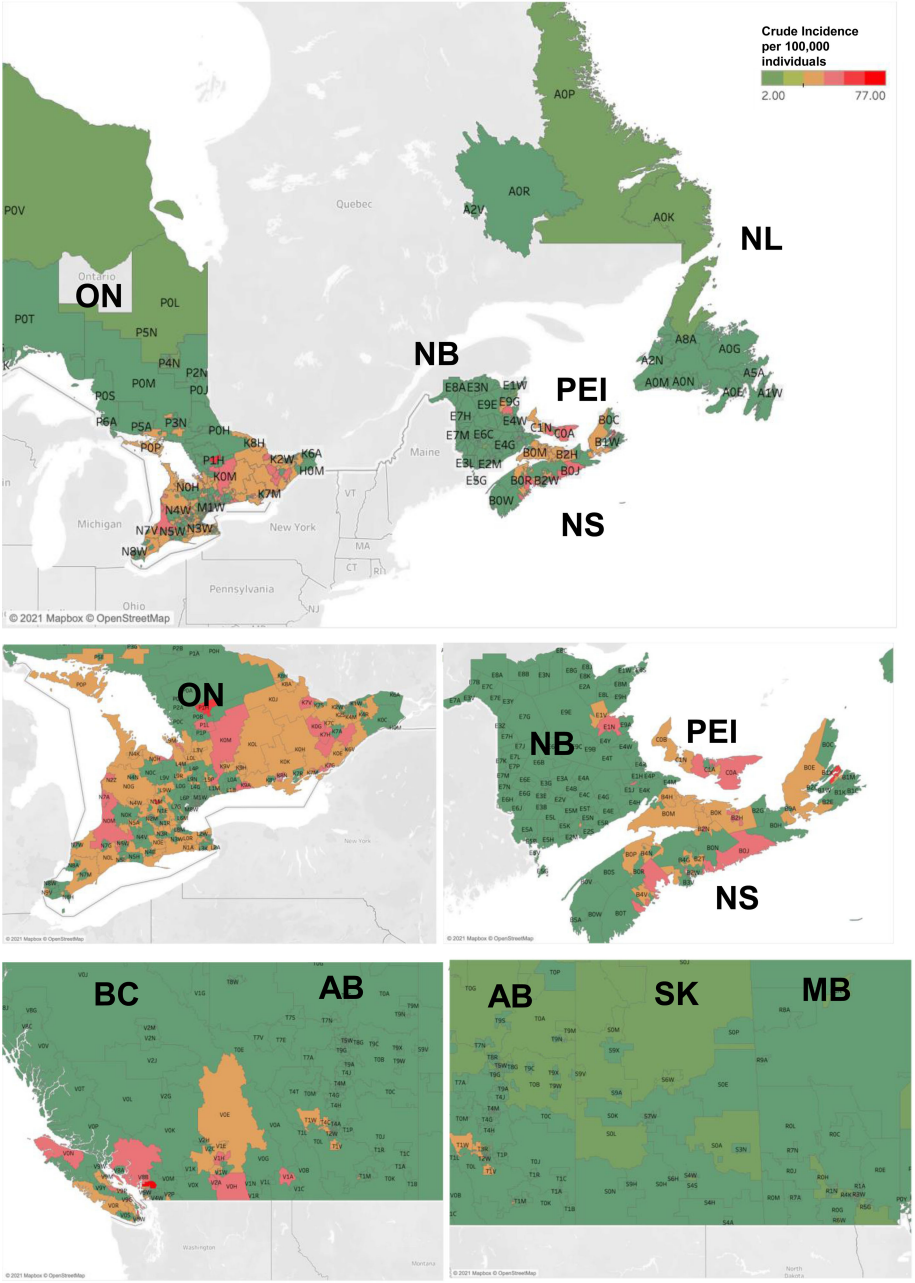
Incidence and mortality rates of CM in Canadian provinces and territories during 2011–2017. Geographic maps illustrating incidence rates of CM (per 100,000 individuals per year) by Forward Sortation Area (FSA) postal code. NL- Newfoundland and Labrador; NS-Nova Scotia, NB-New Brunswick; PEI- Prince Edward Island; QC-Quebec; ON-Ontario; MB-Manitoba, SK-Saskatchewan; AB-Alberta; BC-British Columbia.

We further analyzed CM incidence rates by postal code [forward sortation area (FSA); the first 3 entries in the postal code – e.g., H4A]. This analysis highlighted that postal codes/FSAs located in the southern regions of Canada, especially in the proximity of warmer waters (southern and coastal British Columbia, southern Ontario, Nova Scotia, Prince Edward Island and New Brunswick communities) as well as certain regions located near well-known vacation areas (e.g., postal codes near Banff National Park, AB) had higher rates of CM (Figure 3 and Supplementary Tables 2, 3). Outside of these high-risk areas select locations in city centers (A1E postal code in St. John's, NL; R2G, R3F and R3P postal codes in Winnipeg, MB; T2V and T2L postal codes in Calgary, AB) were also noted to have higher CM incidence (Figure 3 and Supplementary Tables 2, 3).

### Analysis of CM Mortality Across Canada

The CVS database enabled us to examine deaths caused by CM (Table 2C). Mortality trends were reflective of the detailed above incidence trends, whereby there were more deaths due to CM in males than in females (62.9 *vs*. 37.1%; *p* < 0.001) and differences were observed by region. Notably, melanoma mortality in both sexes since 2013 has been decreasing in Canada (Figure 1A). Analysis by age group found that deaths peaked in the 70–80's age group (Table 2C).

## Discussion

Using the CCR and CVS databases, this paper provides a detailed description of the epidemiologic trends of CM in Canada between 2011 and 2017 highlighting variation by age, sex, anatomic site involved as well as striking geographic differences. While CM is increasing in Canada at an alarming rate, based on the mapping analysis, it is evident that southern regions of Ontario, British Columbia, and maritime provinces, especially, when surrounded by warmer waters, had significantly higher incidence rates. These results also correlate well with our and other previous reports highlighting variation in population by province/territory by Fitzpatrick skin phototype in Canada (12, 35). Specifically, the provinces of high CM incidence (NS and PEI) had the highest percentage of individuals with a British Isles background, which includes people of Cornish, English, Irish, Manx, Scottish and Welsh descent, who are known to have a predominance of Fitzpatrick skin types I-II (12, 35).

Notably, previous reports comparing coastal *vs*. inland areas documented that there was a greater incidence of CM cases along the coast, with an IRR of 1.23 after adjusting for socioeconomic status, UV index and latitude (36). Our mapping results visually underscore these findings in Canada, while providing specific details on the communities at risk. Hence, CM incidence in high-risk population (e.g., Fitzpatrick phototype I-III skin) greatly depends on climate/geography, which impacts human behavior, clothing styles and sun protection practices leading to higher or lower melanoma rates. Our findings are also in-line with the recently reported melanoma analysis on NL highlighting similar trends (35).

Our study highlights that CM incidence rates continue to increase in both sexes, while CM mortality is declining since 2013 likely due to the emergence of effective targeted and immunotherapy treatments (37). The overall incidence rate of CM in Canada was 20.75 cases per 100,000 individuals per year (22.70 in males and 18.84 in females) and the ASIR per 100,000 in Canada was 14.12 cases per 100,000 individuals per year. In 2017, the United States' Surveillance, Epidemiology, and End Results Program (SEER) database found the ASIR of melanoma to be 30.1 cases per 100,000 males, and 18.5 cases per 100,000 females across all ethnicities (38). When observing the United States ASIR trends for 2000–2018, a steady increase in incidence in both sexes was noted, similar to the evolution of CM in Canada, although the rate of change for males was more pronounced. Considering the north-to-south gradient that has been previously established (10), it is expected that the United States, with a great majority of its population living in warmer, sunnier climates has higher rates of CM than Canada. Furthermore, as expected, warmer countries with substantial population comprised of fair skin individuals have even higher rates of CM. The 2012 CM ASIRs for New Zealand and Australia were 35.8 and 34.9 cases per 100,000 individuals per year, respectively (39).

Ultraviolet radiation is the primary risk factor for CM, with sunlight acting as the main source for UV rays, along with artificial sources, such as tanning beds, booths or sun lamps (40–45). We noted an increase in incidence in all anatomic sites, with the male trunk and female extremities showing the most significant increases in incidence. Notably, out of all CM subtypes studied, acral lentiginous melanoma was the only one demonstrating a female predominance (56.9 *vs*. 43.1% in males) and was previously hypothesized to have been associated with increased exposure to UV nail lamps (at times in individuals taking a photosensitizing medication) (46–48). Considering this, in addition to targeting high risk geographic areas, it is of utmost importance to tailor recommendations for each sex/gender differently. According to the American Cancer Society, melanoma of the trunk and legs have the highest association to frequent sunburns, when compared to other sites (49). For this reason, public education campaigns targeting to protect high-risk anatomic sites in different sexes/genders are essential in preventing CM.

CM represents a significant patient and economic burden. Not only is it among the most common cancers found in adolescent and young adult populations, but it is also one of the leading skin cancers in terms of average years of life lost due to a disease (50). On average, an individual dying from melanoma loses 20.4 years of potential life (51). In 2015, melanoma was responsible for almost 1.6 million disease-adjusted life years (DALY) globally, which, when age-standardized, gave a rate of 23 DALYs per 100,000 individuals (52). The total estimated cost to the Canadian healthcare system of skin cancer in 2004 was $532 million, almost 85% of which was attributable to costs related to melanoma treatment (53). Projections concluded that by 2030's in Canada, the financial burden of skin cancer would rise to $1 billion annually, with melanoma consuming the greatest majority of these resources (53).

## Conclusions

Effective programs to help decrease UV radiation exposure have been conducted. For instance, in Australia, a country with one of the highest incidence of CM in the world (54), due to effective public health campaigns, the proportion of adolescents/young adults who reported preferring having a tan decreased from 60% in 2003 to 38% in 2019 (55–57). Development of an effective campaign requires detailed knowledge of the population at risk, awareness levels, potential specific risk factors contributing to melanoma incidence and barriers to sun protection practices. This study represents an important step toward refinement of melanoma/skin cancer campaigns in Canada and provides important epidemiologic data for this vast and multicultural region of the world.

## Limitations

Due to the nature of large, population-based studies, this retrospective analysis had several limitations including missing data and a risk of misclassification of patients (58). An important limitation was unavailability of data for Quebec during this period. Finally, this study could not explore confounding factors that influence melanoma incidence and mortality, including but not limited to ethnicity, clinical staging or Breslow thickness and patient socioeconomic status because this data was not available.

It is important to highlight that as Canada's healthcare system is a single-tier (payer), funded and operated by the provincial governments, the data is collected with consistency, where each provincial and territorial cancer registry identifies tumors in its population by combining information from multiple sources (cancer clinic files, radiotherapy and hematology reports, records from in-patient hospital stays, out-patient clinics, pathology and other laboratory/autopsy reports, radiology/screening program reports, medical billing and hospital discharge administrative databases). The CCR performs multiple processes to ensure accuracy including an internal record linkage to identify possible duplicate records.

Several studies investigated the detection rates/accuracy of diagnostic data in the largest provincial branch of the CCR: the Ontario Cancer Registry which collects data from the most populous province. In fact, a case ascertainment of ~99%, a detection rate (detecting and accurately assigning index tumor site) of 81.4–96%, and a confirmation rate (correctly assigning tumor site) of 90.9% were documents by several studies (59–61), which confirms the high quality of data and detection rates in the examined registries.

## Data Availability Statement

The original contributions presented in the study are included in the article/Supplementary Material, further inquiries can be directed to the corresponding author/s.

## Author Contributions

SC and FG collected data, plotted and analyzed data, plotted the figures, and wrote the initial draft of the manuscript. ML and AA collected data. FL, IM, JC, AM, WM, JC, TS, EN, and RG analyzed data, prepared tables and figures, and co-wrote the manuscript. HN and ER performed statistical analyses. DS collected data, performed statistical analyses, and wrote the article. IL collected data, obtained funding, designed and supervised the study, and wrote the article. All authors contributed to the article and approved the submitted version.

## Funding

This work was supported by the Canadian Institutes for Health Research (CIHR) Project Scheme Grant #426655 to IL, CIHR Catalyst Grant #428712 to IL, FG, ML, FL, IM, JC, AM, JC, EN, RG, DS, and ER; Cancer Research Society (CRS)-CIHR Partnership Grant #25343 to IL; Canadian Dermatology Foundation research grants to IL and DS, and by the Fonds de la recherche du Québec – Santé to IL (#34753, #36769, and #296643).

## Conflict of Interest

The authors declare that the research was conducted in the absence of any commercial or financial relationships that could be construed as a potential conflict of interest.

## Publisher's Note

All claims expressed in this article are solely those of the authors and do not necessarily represent those of their affiliated organizations, or those of the publisher, the editors and the reviewers. Any product that may be evaluated in this article, or claim that may be made by its manufacturer, is not guaranteed or endorsed by the publisher.

## References

[1] Iglesias-Pena N Paradela S Tejera-Vaquerizo A Boada A Fonseca E. Cutaneous melanoma in the elderly: review of a growing problem. Actas Dermosifiliogr. (2019) 110:434–47. 10.1016/j.adengl.2019.05.012 31101317

[2] Canada, PHAo,. Melanoma Skin Cancer. Available online at: http://www.canada.ca/en/public-health/services/chronic-diseases/cancer/melanoma-skincancer.html (accessed June 29, 2021).

[3] Advocacy GCfMP. Melanoma Skin Cancer Report: Stemming the Global Epidemic (2020).

[4] Whiteman D Green A. Chapter 1.2 Epidemiology of Malignant Melanoma. Skin Cancer – A World-Wide Perspective. Berlin: Spring-Verlag (2011). 10.1007/978-3-642-05072-5_2

[5] Rastrelli M Tropea S Rossi CR Alaibac M. Melanoma: epidemiology, risk factors, pathogenesis, diagnosis and classification. In Vivo. (2014) 28:1005–11. 25398793

[6] Staff, MC,. Melanoma - Symptoms and Causes. Available online at: https://www.mayoclinic.org/diseases-conditions/melanoma/symptoms-causes/syc-20374884 (accessed June 29, 2021).

[7] Mc Aleer MA Lacey N Powell FC. The pathophysiology of rosacea. G Ital Dermatol Venereol. (2009) 144:663–71. 19907405

[8] Singer S Karrer S Berneburg M. Modern sun protection. Curr Opin Pharmacol. (2019) 46:24–8. 10.1016/j.coph.2018.12.006 30731327

[9] Wang Z Lu F Li X Guo Y Li J He L. Chinese women with melasma exhibit a low minimal erythema dose to both UVA and UVB. Photodermatol Photoimmunol Photomed. (2021) 93:519–30. 10.1111/phpp.12713 34171129

[10] Ghazawi FM Cyr J Darwich R Le M Rahme E Moreau L. Cutaneous malignant melanoma incidence and mortality trends in Canada: A comprehensive population-based study. J Am Acad Dermatol. (2019) 80:448–59. 10.1016/j.jaad.2018.07.041 30092328

[11] Ghazawi FM Le M Alghazawi N Rahme E Moreau L Netchiporouk E. Trends in incidence of cutaneous malignant melanoma in Canada: 1992-2010 versus 2011-2015. J Am Acad Dermatol. (2019) 80:1157–9. 10.1016/j.jaad.2018.10.055 30395916

[12] Ghazawi FM Le M Lagacé F Cyr J Alghazawi N Zubarev A. Incidence, mortality, and spatiotemporal distribution of cutaneous malignant melanoma cases across Canada. J Cutan Med Surg. (2019) 23:394–412. 10.1177/1203475419852048 31132871

[13] Darwich R Ghazawi FM Rahme E Alghazawi N Zubarev A Moreau L. Epidemiology of ophthalmic lymphoma in Canada during 1992-2010. Br J Ophthalmol. (2020) 104:1176–80. 10.1136/bjophthalmol-2019-314653 31722877

[14] Ghazawi FM Le M Cyr J Netchiporouk E Rahme E Alakel A. Analysis of acute myeloid leukemia incidence and geographic distribution in Canada from 1992 to 2010 reveals disease clusters in Sarnia and other industrial US border cities in Ontario. Cancer. (2019) 125:1886–97. 10.1002/cncr.32034 30811592

[15] Ghazawi FM Lu J Savin E Zubarev A Chauvin P Sasseville D. Epidemiology and patient distribution of oral cavity and oropharyngeal SCC in Canada. J Cutan Med Surg. (2020) 24:340–9. 10.1177/1203475420915448 32238063

[16] Ghazawi FM Ramanakumar AV Alakel A Lagacé F Chen A Le M. Incidence of acute myeloid leukemia: A regional analysis of Canada. Cancer. (2020) 126:1356–61. 10.1002/cncr.32671 31873963

[17] Menter A Strober BE Kaplan DH Kivelevitch D Prater EF Stoff B. Joint AAD-NPF guidelines of care for the management and treatment of psoriasis with biologics. J Am Acad Dermatol. (2019) 80:1029–72. 10.1016/j.jaad.2018.11.057 30772098

[18] Le M Ghazawi FM Rahme E Alakel A Netchiporouk E Savin E. Identification of significant geographic clustering of polycythemia vera cases in Montreal, Canada. Cancer. (2019) 125:3953–9. 10.1002/cncr.32417 31381139

[19] Tsang M Le M Ghazawi FM Cyr J Alakel A Rahme E. Multiple myeloma epidemiology and patient geographic distribution in Canada: A population study. Cancer. (2019) 125:2435–44. 10.1002/cncr.32128 30951209

[20] Ghazawi FM Darwich R Le M Jfri A Rahme E Valdemarin Burnier J. Incidence trends of conjunctival malignant melanoma in Canada. Br J Ophthalmol. (2020) 104:23–5. 10.1136/bjophthalmol-2019-313977 31079055

[21] Ghazawi FM Darwich R Le M Rahme E Zubarev A Moreau L. Uveal melanoma incidence trends in Canada: a national comprehensive population-based study. Br J Ophthalmol. (2019) 103:1872–6. 10.1136/bjophthalmol-2018-312966 30819691

[22] Ghazawi FM Netchiporouk E Rahme E Tsang M Moreau L Glassman S. Comprehensive analysis of cutaneous T-cell lymphoma (CTCL) incidence and mortality in Canada reveals changing trends and geographic clustering for this malignancy. Cancer. (2017) 123:3550–67. 10.1002/cncr.30758 28493286

[23] Ghazawi FM Netchiporouk E Rahme E Tsang M Moreau L Glassman S. Distribution and clustering of cutaneous T-cell lymphoma (CTCL) cases in Canada during 1992 to 2010. J Cutan Med Surg. (2017) 2017:1203475417745825. 10.1177/1203475417745825 29241349

[24] Cattelan L Ghazawi FM Le M Lagacé F Savin E Zubarev A. Epidemiologic trends and geographic distribution of esophageal cancer in Canada: A national population-based study. Cancer Med. (2020) 9:401–17. 10.1002/cam4.2700 31715645 PMC6943153

[25] Cattelan L Ghazawi FM Le M Savin E Zubarev A Lagacé F. Investigating epidemiologic trends and the geographic distribution of patients with anal squamous cell carcinoma throughout Canada. Curr Oncol. (2020) 27:e294–306. 10.3747/co.27.6061 32669936 PMC7339845

[26] Darwich R Ghazawi FM Le M Rahme E Alghazawi N Zubarev A. Epidemiology of invasive ocular surface squamous neoplasia in Canada during 1992-2010. Br J Ophthalmol. (2020) 104:1368–72. 10.1136/bjophthalmol-2019-314650 31949098

[27] Darwich R Ghazawi FM Rahme E Alghazawi N Valdemarin Burnier J Sasseville D. Retinoblastoma incidence trends in Canada: a national comprehensive population-based study. J Pediatr Ophthalmol Strabismus. (2019) 56:124–30. 10.3928/01913913-20190128-02 30889267

[28] Lagacé F Ghazawi FM Le M Rahme E Savin E Zubarev A. Analysis of incidence, mortality trends, and geographic distribution of breast cancer patients in Canada. Breast Cancer Res Treat. (2019) 178:683–91. 10.1007/s10549-019-05418-2 31485819

[29] Lagacé F Ghazawi FM Le M Savin E Zubarev A Powell M. Penile invasive squamous cell carcinoma: analysis of incidence, mortality trends, and geographic distribution in Canada. J Cutan Med Surg. (2020) 24:124–8. 10.1177/1203475419888869 31722549

[30] Lagacé F Ghazawi FM Le M Savin E Zubarev A Powell M. Incidence and mortality of prostate cancer in Canada during 1992-2010. Curr Oncol. (2021) 28:978–90. 10.3390/curroncol28010096 33617514 PMC7985768

[31] Le M Ghazawi FM Alakel A Netchiporouk E Rahme E Zubarev A. Incidence and mortality trends and geographic patterns of follicular lymphoma in Canada. Curr Oncol. (2019) 26:e473–81. 10.3747/co.26.4625 31548815 PMC6726266

[32] Roy SF Ghazawi FM Le M Lagacé F Roy CF Rahme E. Epidemiology of adult and pediatric Burkitt lymphoma in Canada: sequelae of the HIV epidemic. Curr Oncol. (2020) 27:83–9. 10.3747/co.27.5775 32489250 PMC7253744

[33] Xiao Y Cattelan L Lagacé F Ghazawi FM Alakel A Grose E. Epidemiologic trends and geographic distribution of patients with gallbladder and extrahepatic biliary tract cancers in Canada. HPB. (2021) 23:1467–628. 10.1016/j.hpb.2021.03.007 33863655

[34] Spiegelman M. Introduction to Demography, Revised Edition - Formula 4.29. (1969).

[35] Gulliver W Gulliver S Power RJ Penney M Lane D. The incidence of cutaneous malignant melanoma in eastern newfoundland and labrador, Canada, from 2007 to 2015. Dermatology. (2021) 2021:1–7. 10.1159/000519193 34610598

[36] Korgavkar K Lee KC Weinstock MA. Higher melanoma incidence in coastal versus inland counties in California. Melanoma Res. (2014) 24:280–5. 10.1097/CMR.0000000000000067 24681542

[37] Nogrady B. Game-changing class of immunotherapy drugs lengthens melanoma survival rates. Nature. (2020) 580:S14–6. 10.1038/d41586-020-01038-9

[38] Institute, NC,. Melanoma of the Skin: Recent Trends in SEER Age-Adjusted Incidence Rates, 2000-2018. Available online at: https://seer.cancer.gov/explorer/application.html?site=53&data_type=1&graph_type=2&compareBy=sex&chk_sex_3=3&chk_sex_2=2&rate_type=2&race=1&age_range=1&stage=101&advopt_precision=1&advopt_show_ci=on&advopt_display=2 (accessed July 2, 2021).

[39] Ward WH Farma JM. Cutaneous Melanoma: Etiology and Therapy. (2017). 10.15586/codon.cutaneousmelanoma.2017 29461771

[40] Gandini S Sera F Cattaruzza MS Pasquini P Picconi O Boyle P. Meta-analysis of risk factors for cutaneous melanoma: II. Sun exposure. Eur J Cancer. (2005) 41:45–60. 10.1016/j.ejca.2004.10.016 15617990

[41] Moan J Cicarma E Setlow R Porojnicu AC Grant WB Juzeniene A. Time trends and latitude dependence of uveal and cutaneous malignant melanoma induced by solar radiation. Dermato-Endocrinol. (2010) 2:3–8. 10.4161/derm.2.1.11745 21547141 PMC3084958

[42] Nikolaou VA Sypsa V Stefanaki I Gogas H Papadopoulos O Polydorou D. Risk associations of melanoma in a southern european population: results of a case/control study. Cancer Causes Control. (2008) 19:671–9. 10.1007/s10552-008-9130-0 18307049

[43] Elwood JM Gallagher RP Davison J Hill GB. Sunburn, suntan and the risk of cutaneous malignant melanoma - The Western Canada Melanoma Study. Br J Cancer. (1985) 51:543–9. 10.1038/bjc.1985.77 3978032 PMC1977145

[44] Veierod MB Adami HO Lund E Armstrong BK Weiderpass E. Sun and solarium exposure and melanoma risk: effects of age, pigmentary characteristics, and nevi. Cancer Epidemiol Biomarkers Prev. (2010) 19:111–20. 10.1158/1055-9965.EPI-09-0567 20056629

[45] Wilson NJ Boniface K Chan JR McKenzie BS Blumenschein WM Mattson JD. Development, cytokine profile and function of human interleukin 17-producing helper T cells. Nat Immunol. (2007) 8:950–7. 10.1038/ni1497 17676044

[46] MacFarlane DF Alonso CA. Occurrence of nonmelanoma skin cancers on the hands after UV nail light exposure. Arch Dermatol. (2009) 145:447–9. 10.1001/archdermatol.2008.622 19380667

[47] Freeman C Hull C Sontheimer R Curtis J. Squamous cell carcinoma of the dorsal hands and feet after repeated exposure to ultraviolet nail lamps. Dermatol Online J. (2020) 26:7974. 10.5070/D3263047974 32609442

[48] O'Sullivan NA Tait CP. Tanning bed and nail lamp use and the risk of cutaneous malignancy: a review of the literature. Australas J Dermatol. (2014) 55:99–106. 10.1111/ajd.12145 24592921

[49] team, TACSmaec,. Risk Factors for Melanoma Skin Cancer. Available online at: https://www.cancer.org/cancer/melanoma-skin-cancer/causes-risks-prevention/risk-factors.html (accessed June 29, 2021).

[50] Matthews NH Cutaneous Melanoma: Etiology and Therapy. In: Ward WH, Farma JM, editors. Brisbane, AU: Codon Publications Copyright © 2017 Codon Publications (2017). 29461771

[51] Guy GP Thomas CC Thompson T Watson M Massetti GM Richardson LC. Vital signs: melanoma incidence and mortality trends and projections - United States, 1982-2030. MMWR Morb Mortal Wkly Rep. (2015)64:591–6. 26042651 PMC4584771

[52] Karimkhani C Green AC Nijsten T Weinstock MA Dellavalle RP Naghavi M. The global burden of melanoma: results from the Global Burden of Disease Study 2015. Br J Dermatol. (2017) 177:134–140. 10.1111/bjd.15510 28369739 PMC5575560

[53] Joshua AM. Melanoma prevention: are we doing enough? A Canadian perspective. Curr Oncol. (2012) 19:e462–7. 10.3747/co.19.1222 23300369 PMC3503676

[54] Liu V Mihm MC. Pathology of malignant melanoma. Surg Clin N Am. (2003) 83:31–60. 10.1016/S0039-6109(03)00003-3 12691449

[55] Horsham C Antrobus J Olsen CM Ford H Abernethy D Hacker E. Testing wearable UV sensors to improve sun protection in young adults at an outdoor festival: field study. JMIR Mhealth Uhealth. (2020) 8:e21243. 10.2196/21243 32936083 PMC7531871

[56] Tabbakh T Volkov A Wakefield M Dobbinson S. Implementation of the SunSmart program and population sun protection behaviour in Melbourne, Australia: Results from crosssectional summer surveys from 1987 to 2017. PLoS Med. (2019) 16:e1002932. 10.1371/journal.pmed.1002932 31593565 PMC6782093

[57] Iannacone MR Green AC. Towards skin cancer prevention and early detection: evolution of skin cancer awareness campaigns in Australia. Melanoma Manag. (2014) 1:75–84. 10.2217/mmt.14.6 30190812 PMC6094686

[58] Sanders CM Saltzstein SL Schultzel MM Nguyen DH Stafford HS Sadler GR. Understanding the limits of large datasets. J Cancer Educ. (2012) 27:664–9. 10.1007/s13187-012-0383-7 22729362 PMC4153382

[59] Greenberg ML Barr RD DiMonte B McLaughlin E Greenberg C. Childhood cancer registries in Ontario, Canada: lessons learned from a comparison of two registries. Int J Cancer. (2003) 105:88–91. 10.1002/ijc.11004 12672035

[60] Gupta S Pole JD. The validity of pediatric cancer diagnoses in a population-based general cancer registry in Ontario, Canada. BMC Cancer. (2016) 16:885. 10.1186/s12885-016-2931-8 27842503 PMC5109739

[61] Hall S Schulze K Groome P Mackillop W Holowaty E. Using cancer registry data for survival studies: the example of the Ontario Cancer Registry. J Clin Epidemiol. (2006) 59:67–76. 10.1016/j.jclinepi.2005.05.001 16360563

