# Supplementary material for: Population-Based Study Detailing Cutaneous Melanoma Incidence and Mortality Trends in Canada
Source: Front Med (Lausanne). 2022 Mar 3;9:830254. doi: 10.3389/fmed.2022.830254 (PMC8927870; doi:10.3389/fmed.2022.830254)
Supplement: Supplementary file 1 [file Data_Sheet_1.PDF]

Supplementary Table 1: (A) Subtypes of CMM that were queried and analyzed in this study, as defined based on the International Classification of Diseases for Oncology, Third Edition (ICD-O-3). (B) Cause of death analysis codes that were queried and analyzed in this study, as defined based on the International Statistical Classification of Diseases and Related Health Problems, 10th Revision (ICD-10).

(A)

**ICD-O-3**	**Name of the corresponding cancer type**
8720	Malignant melanoma, Not Otherwise Specified (NOS)
8721	Nodular melanoma
8723	Malignant melanoma, regressing
8730	Amelanotic melanoma
8740	Malignant melanoma in junctional nevus
8742	Lentigo maligna melanoma
8743	Superficial spreading melanoma
8744	Acral lentiginous melanoma
8745	Desmoplastic melanoma
8761	Malignant melanoma in a giant pigmented nevus

(B)

**ICD-10**	**Name of the corresponding cause of death**
C43.2	Malignant melanoma of ear and external auricular canal
C43.3	Malignant melanoma of other and unspecified parts of face
C43.4	Malignant melanoma of scalp and neck
C43.5	Malignant melanoma of trunk
C43.6	Malignant melanoma of upper limb, including shoulder
C43.7	Malignant melanoma of lower limb, including hip
C43.9	Malignant melanoma of skin, unspecified

Supplementary Table 2: List of 210 statistically significant high-incidence FSAs and their corresponding population sizes. All population numbers are rounded to the nearest thousand.

FSA	Cases	Population, Rounded	Incidence per 100,000 individuals per year, Rounded	Lower CI (95%), Rounded	Upper CI (95%), Rounded	Province
K0K	275	110000	35.61	31.52	40.07	Eastern Ontario
V0N	225	78000	41.26	36.05	47.02	British Columbia
K0A	215	101000	30.31	26.4	34.65	Eastern Ontario
N0M	205	65000	45.33	39.33	51.98	Southwestern Ontario
L0R	190	93000	29.28	25.27	33.76	Central Ontario
K0L	170	70000	34.64	29.63	40.26	Eastern Ontario
V0H	170	53000	46.17	39.49	53.65	British Columbia
N0G	165	83000	28.42	24.25	33.1	Southwestern Ontario
N0B	155	78000	28.5	24.19	33.35	Southwestern Ontario
K0M	150	49000	44.09	37.32	51.74	Eastern Ontario
L0S	145	53000	38.76	32.7	45.6	Central Ontario
V0E	140	65000	30.73	25.85	36.26	British Columbia
V0R	140	65000	30.8	25.91	36.35	British Columbia
C0A	125	44000	40.64	33.82	48.42	Prince Edward Island
B0J	115	40000	41.44	34.21	49.74	Nova Scotia
K7M	115	47000	34.8	28.73	41.77	Eastern Ontario
N0H	115	47000	34.61	28.58	41.55	Southwestern Ontario
N0L	110	48000	32.98	27.1	39.75	Southwestern Ontario
N6H	110	40000	39.78	32.69	47.94	Southwestern Ontario
V2A	110	37000	42.44	34.88	51.15	British Columbia
K0G	105	36000	41.59	34.02	50.35	Eastern Ontario
L4M	105	45000	33.22	27.17	40.22	Central Ontario
N0N	105	41000	36.83	30.12	44.58	Southwestern Ontario
N0P	105	53000	28.49	23.3	34.49	Southwestern Ontario
V4A	105	36000	41.43	33.89	50.16	British Columbia
K0H	100	44000	32.59	26.52	39.64	Eastern Ontario
L3Y	100	45000	31.84	25.91	38.73	Central Ontario
V2R	100	46000	30.82	25.08	37.49	British Columbia
K9J	95	44000	30.91	25.01	37.78	Eastern Ontario
L0L	95	46000	29.76	24.07	36.37	Central Ontario
L3V	95	44000	30.68	24.82	37.5	Central Ontario
T2V	95	33000	41.27	33.39	50.45	Alberta
K0E	90	39000	33.25	26.74	40.88	Eastern Ontario
L1G	90	41000	31.37	25.23	38.56	Central Ontario
N3R	90	34000	37.51	30.16	46.11	Southwestern Ontario
N6K	90	34000	37.82	30.41	46.49	Southwestern Ontario
V9A	90	37000	34.56	27.79	42.48	British Columbia
B0K	85	36000	33.4	26.68	41.3	Nova Scotia

FSA	Cases	Population, Rounded	Incidence per 100,000 individuals per year, Rounded	Lower CI (95%), Rounded	Upper CI (95%), Rounded	Province
L0K	85	34000	35.3	28.2	43.65	Central Ontario
L9W	85	41000	29.73	23.75	36.77	Central Ontario
N7S	85	28000	43.3	34.59	53.54	Southwestern Ontario
T1K	85	45000	27.17	21.7	33.59	Alberta
V8L	85	23000	52.55	41.98	64.98	British Columbia
V9B	85	42000	29.11	23.25	36	British Columbia
B0P	80	40000	28.37	22.5	35.31	Nova Scotia
B2W	80	32000	35.53	28.18	44.23	Nova Scotia
K0J	80	34000	33.98	26.94	42.29	Eastern Ontario
K8N	80	28000	41.05	32.55	51.09	Eastern Ontario
L1S	80	41000	28.15	22.32	35.04	Central Ontario
L2N	80	31000	37.03	29.36	46.08	Central Ontario
L6L	80	29000	39.14	31.03	48.71	Central Ontario
L9H	80	31000	36.45	28.9	45.36	Central Ontario
L9Y	80	23000	49.21	39.02	61.24	Central Ontario
N0E	80	35000	32.88	26.07	40.92	Southwestern Ontario
V1W	80	33000	34.51	27.36	42.95	British Columbia
K1C	75	38000	28.32	22.28	35.5	Eastern Ontario
K9A	75	25000	42.73	33.61	53.57	Eastern Ontario
L2M	75	33000	32.82	25.82	41.14	Central Ontario
N0A	75	33000	32.58	25.63	40.84	Southwestern Ontario
N5A	75	31000	34.46	27.1	43.2	Southwestern Ontario
V8V	75	25000	42.76	33.63	53.6	British Columbia
V9L	75	34000	31.4	24.7	39.37	British Columbia
V9N	75	29000	36.51	28.72	45.76	British Columbia
V9P	75	25000	43.27	34.03	54.24	British Columbia
A1E	70	28000	36.06	28.11	45.55	Newfoundland and Labrador
V7V	70	15000	66.97	52.21	84.61	British Columbia
B3M	65	31000	30.28	23.37	38.6	Nova Scotia
C1A	65	27000	34.27	26.45	43.68	Prince Edward Island
K2S	65	30000	30.55	23.58	38.94	Eastern Ontario
K7L	65	19000	48.85	37.7	62.26	Eastern Ontario
K8A	65	26000	35.41	27.33	45.14	Eastern Ontario
K9H	65	28000	33.16	25.59	42.27	Eastern Ontario
K9V	65	27000	34.23	26.42	43.63	Eastern Ontario
N5R	65	27000	34.19	26.38	43.57	Southwestern Ontario
N5X	65	31000	29.81	23.01	37.99	Southwestern Ontario
N6J	65	27000	34.42	26.57	43.87	Southwestern Ontario
R2G	65	33000	28.29	21.83	36.06	Manitoba
T1S	65	31000	30.1	23.23	38.37	Alberta
V1Y	65	33000	28.29	21.83	36.06	British Columbia
V8S	65	17000	54.97	42.42	70.06	British Columbia

FSA	Cases	Population, Rounded	Incidence per 100,000 individuals per year, Rounded	Lower CI (95%), Rounded	Upper CI (95%), Rounded	Province
B0E	60	26000	32.69	24.95	42.08	Nova Scotia
B2N	60	20000	42.88	32.73	55.2	Nova Scotia
B3H	60	19000	45.53	34.74	58.6	Nova Scotia
C0B	60	29000	29.56	22.56	38.06	Prince Edward Island
K2H	60	26000	32.5	24.8	41.84	Eastern Ontario
L1H	60	31000	27.46	20.96	35.35	Central Ontario
L4R	60	18000	47.53	36.27	61.19	Central Ontario
N1M	60	16000	55.02	41.98	70.82	Southwestern Ontario
N4K	60	29000	30.04	22.93	38.67	Southwestern Ontario
N6C	60	31000	27.84	21.24	35.83	Southwestern Ontario
P1H	60	16000	53.23	40.62	68.52	Northern Ontario
P3E	60	27000	31.68	24.17	40.78	Northern Ontario
V1B	60	21000	40.95	31.25	52.71	British Columbia
V8X	60	23000	37.28	28.45	47.98	British Columbia
V8Z	60	30000	28.36	21.64	36.5	British Columbia
B4V	55	20000	39.4	29.68	51.29	Nova Scotia
E1B	55	19000	40.5	30.51	52.72	New Brunswick
K6V	55	28000	28.02	21.11	36.47	Eastern Ontario
L6J	55	25000	31.73	23.91	41.31	Central Ontario
L7N	55	13000	60.76	45.77	79.08	Central Ontario
L9Z	55	19000	41.14	30.99	53.55	Central Ontario
M4N	55	15000	51.51	38.81	67.05	Metropolitan Toronto
N1G	55	27000	29.55	22.26	38.47	Southwestern Ontario
R3R	55	25000	31.58	23.79	41.11	Manitoba
T4C	55	26000	29.71	22.38	38.67	Alberta
V1T	55	28000	28.52	21.49	37.13	British Columbia
V1V	55	20000	39.3	29.61	51.16	British Columbia
V4B	55	20000	40.01	30.14	52.08	British Columbia
V8M	55	16000	49.66	37.41	64.64	British Columbia
B2H	50	15000	47.91	35.56	63.16	Nova Scotia
B4A	50	18000	40.35	29.95	53.2	Nova Scotia
K7H	50	15000	47.51	35.26	62.63	Eastern Ontario
L1A	50	15000	47.41	35.19	62.51	Central Ontario
L9A	50	25000	28.97	21.5	38.2	Central Ontario
L9B	50	22000	31.85	23.64	41.98	Central Ontario
N3Y	50	22000	32.11	23.83	42.33	Southwestern Ontario
N4G	50	19000	37.37	27.74	49.27	Southwestern Ontario
N7L	50	25000	28.82	21.39	37.99	Southwestern Ontario
N9V	50	20000	35.67	26.47	47.02	Southwestern Ontario
P1L	50	16000	43.78	32.49	57.72	Northern Ontario
V4K	50	24000	29.25	21.71	38.56	British Columbia
V8R	50	23000	30.72	22.8	40.5	British Columbia

FSA	Cases	Population, Rounded	Incidence per 100,000 individuals per year, Rounded	Lower CI (95%), Rounded	Upper CI (95%), Rounded	Province
B0M	45	17000	37.04	27.02	49.56	Nova Scotia
B2T	45	17000	38.75	28.26	51.85	Nova Scotia
B3Z	45	17000	38.88	28.36	52.03	Nova Scotia
K7P	45	20000	31.62	23.06	42.31	Eastern Ontario
L8V	45	21000	30.24	22.06	40.46	Central Ontario
L9L	45	15000	43.5	31.73	58.21	Central Ontario
L9R	45	19000	33.19	24.21	44.41	Central Ontario
M4V	45	18000	36.19	26.4	48.42	Metropolitan Toronto
N7V	45	12000	53.86	39.29	72.07	Southwestern Ontario
R3P	45	19000	33.58	24.49	44.93	Manitoba
V1H	45	13000	49.7	36.25	66.5	British Columbia
V6J	45	22000	29.62	21.61	39.64	British Columbia
V8Y	45	10000	65.22	47.57	87.26	British Columbia
V9K	45	15000	42.04	30.67	56.26	British Columbia
B3L	40	17000	32.71	23.37	44.54	Nova Scotia
B4E	40	18000	32.44	23.18	44.18	Nova Scotia
B4N	40	17000	32.68	23.35	44.5	Nova Scotia
C1N	40	16000	34.82	24.88	47.42	Prince Edward Island
E1N	40	12000	49.47	35.35	67.37	New Brunswick
E2E	40	19000	29.34	20.96	39.95	New Brunswick
K1Y	40	19000	29.33	20.96	39.95	Eastern Ontario
K2A	40	16000	34.98	24.99	47.63	Eastern Ontario
K7C	40	17000	33.72	24.09	45.92	Eastern Ontario
K7V	40	13000	42.72	30.52	58.17	Eastern Ontario
L8T	40	19000	29.92	21.38	40.74	Central Ontario
L9M	40	15000	39.36	28.12	53.6	Central Ontario
L9P	40	17000	34.12	24.38	46.46	Central Ontario
M5P	40	19000	30.27	21.62	41.21	Metropolitan Toronto
N2B	40	17000	34.28	24.49	46.68	Southwestern Ontario
N2J	40	18000	31.79	22.72	43.3	Southwestern Ontario
P0P	40	19000	29.7	21.22	40.44	Northern Ontario
V1E	40	19000	29.71	21.22	40.45	British Columbia
V7J	40	16000	35.14	25.11	47.86	British Columbia
V7N	40	15000	38.43	27.45	52.33	British Columbia
V7R	40	15000	38.45	27.47	52.36	British Columbia
V7W	40	9000	66.37	47.41	90.37	British Columbia
V8P	40	18000	31.95	22.83	43.51	British Columbia
V9G	40	14000	39.67	28.34	54.02	British Columbia
V9M	40	17000	33.72	24.09	45.91	British Columbia
B3S	35	12000	42.33	29.48	58.87	Nova Scotia
B4H	35	14000	36.48	25.41	50.74	Nova Scotia
B6L	35	13000	38.67	26.94	53.78	Nova Scotia

FSA	Cases	Population, Rounded	Incidence per 100,000 individuals per year, Rounded	Lower CI (95%), Rounded	Upper CI (95%), Rounded	Province
L7R	35	16000	30.84	21.48	42.89	Central Ontario
M4W	35	14000	35	24.38	48.67	Metropolitan Toronto
N2Z	35	11000	46.76	32.57	65.03	Southwestern Ontario
N3L	35	14000	34.71	24.18	48.27	Southwestern Ontario
N7A	35	12000	42.74	29.77	59.45	Southwestern Ontario
T1V	35	15000	32.4	22.57	45.06	Alberta
V1Z	35	14000	36.04	25.11	50.13	British Columbia
V4M	35	14000	34.7	24.17	48.26	British Columbia
V7G	35	12000	42.86	29.86	59.61	British Columbia
V7H	35	13000	39.01	27.17	54.25	British Columbia
V7K	35	12000	40.46	28.18	56.27	British Columbia
V9Z	35	17000	30.23	21.06	42.04	British Columbia
B2V	30	12000	34.53	23.3	49.3	Nova Scotia
B2X	30	12000	36.44	24.59	52.03	Nova Scotia
C1B	30	11000	39.43	26.6	56.28	Prince Edward Island
E1V	30	14000	31.63	21.34	45.15	New Brunswick
K7G	30	8000	51.07	34.45	72.9	Eastern Ontario
L1B	30	11000	37.68	25.43	53.8	Central Ontario
L6K	30	13000	33.79	22.8	48.24	Central Ontario
M4T	30	10000	41.7	28.14	59.53	Metropolitan Toronto
M8X	30	11000	40.31	27.2	57.54	Metropolitan Toronto
N1A	30	12000	35.95	24.26	51.33	Southwestern Ontario
N4W	30	11000	39.47	26.63	56.35	Southwestern Ontario
N8P	30	11000	37.33	25.19	53.29	Southwestern Ontario
T1W	30	14000	31.24	21.08	44.6	Alberta
T2L	30	14000	31.63	21.34	45.15	Alberta
V4L	30	8000	54.42	36.72	77.69	British Columbia
V4P	30	12000	35.22	23.76	50.27	British Columbia
B1S	25	9000	41.24	26.69	60.88	Nova Scotia
B1Y	25	6000	59.18	38.3	87.36	Nova Scotia
K4M	25	10000	35.35	22.88	52.19	Eastern Ontario
L2T	25	10000	34.59	22.39	51.06	Central Ontario
N4T	25	10000	36.32	23.51	53.62	Southwestern Ontario
N4X	25	10000	37.37	24.18	55.16	Southwestern Ontario
N6A	25	11000	33.45	21.65	49.38	Southwestern Ontario
N6P	25	8000	44.74	28.95	66.05	Southwestern Ontario
T3Z	25	11000	33.06	21.4	48.81	Alberta
V1A	25	8000	44.7	28.93	65.98	British Columbia
V7S	25	11000	33.28	21.54	49.12	British Columbia
V7T	25	9000	38.84	25.14	57.34	British Columbia
B2A	20	8000	35.06	21.41	54.14	Nova Scotia
K1M	20	7000	42.42	25.91	65.52	Eastern Ontario

FSA	Cases	Population, Rounded	Incidence per 100,000 individuals per year, Rounded	Lower CI (95%), Rounded	Upper CI (95%), Rounded	Province
K9L	20	7000	41.73	25.49	64.44	Eastern Ontario
L0P	20	8000	34.33	20.97	53.02	Central Ontario
M2P	20	8000	36.5	22.3	56.37	Metropolitan Toronto
N4L	20	8000	35.49	21.68	54.82	Southwestern Ontario

Supplementary Table 3: List of 130 statistically significant low-incidence FSAs and their corresponding population sizes. All population numbers are rounded to the nearest thousand.

FSA	Cases	Population, Rounded	Incidence per 100,000 individuals per year, Rounded	Lower CI (95%), Rounded	Upper CI (95%), Rounded	Province
V3S	110	100000	15.64	12.86	18.86	British Columbia
L9T	70	91000	10.94	8.53	13.82	Central Ontario
T3K	70	77000	13.04	10.16	16.47	Alberta
T5T	70	64000	15.51	12.09	19.6	Alberta
L5M	65	102000	9.12	7.04	11.62	Central Ontario
L5N	65	85000	10.93	8.44	13.93	Central Ontario
L6H	65	60000	15.37	11.86	19.59	Central Ontario
T2Y	65	60000	15.57	12.02	19.84	Alberta
T0B	60	63000	13.53	10.32	17.41	Alberta
K4A	55	51000	15.45	11.64	20.11	Eastern Ontario
V3B	55	53000	14.77	11.13	19.23	British Columbia
L4L	50	55000	13.02	9.67	17.17	Central Ontario
L6S	50	54000	13.25	9.84	17.47	Central Ontario
T0H	50	83000	8.6	6.39	11.34	Alberta
V2T	50	55000	13.11	9.73	17.28	British Columbia
V4N	50	74000	9.71	7.21	12.81	British Columbia
L5B	45	61000	10.47	7.64	14.01	Central Ontario
L6A	45	81000	7.89	5.76	10.56	Central Ontario
M1E	45	47000	13.78	10.05	18.43	Metropolitan Toronto
S0A	45	53000	12.08	8.81	16.17	Saskatchewan
T0A	45	56000	11.4	8.31	15.25	Alberta
V0J	45	53000	12.07	8.81	16.16	British Columbia
L4E	40	48000	11.82	8.45	16.1	Central Ontario
M2J	40	56000	10.17	7.26	13.85	Metropolitan Toronto
R0A	40	43000	13.34	9.53	18.17	Manitoba
T0E	40	43000	13.38	9.56	18.22	Alberta
T4B	40	41000	13.85	9.89	18.86	Alberta
T7X	40	39000	14.48	10.34	19.72	Alberta
V3J	40	40000	14.19	10.13	19.32	British Columbia

FSA	Cases	Population, Rounded	Incidence per 100,000 individuals per year, Rounded	Lower CI (95%), Rounded	Upper CI (95%), Rounded	Province
L1T	35	49000	10.17	7.08	14.14	Central Ontario
L3R	35	62000	8.02	5.59	11.16	Central Ontario
L6Y	35	81000	6.17	4.3	8.59	Central Ontario
M2N	35	72000	6.99	4.87	9.72	Metropolitan Toronto
R2V	35	35000	14.45	10.07	20.1	Manitoba
S0J	35	49000	10.12	7.05	14.07	Saskatchewan
T1Y	35	56000	8.95	6.23	12.44	Alberta
T2A	35	58000	8.58	5.98	11.93	Alberta
T4R	35	35000	14.24	9.92	19.8	Alberta
T5X	35	39000	12.67	8.83	17.63	Alberta
V3M	35	42000	11.96	8.33	16.64	British Columbia
V3W	35	91000	5.51	3.84	7.66	British Columbia
V4C	35	43000	11.63	8.1	16.17	British Columbia
L3Z	30	30000	14.24	9.61	20.33	Central Ontario
L5A	30	48000	9.02	6.08	12.87	Central Ontario
L6T	30	38000	11.29	7.62	16.11	Central Ontario
M1K	30	48000	8.96	6.04	12.78	Metropolitan Toronto
M1P	30	44000	9.65	6.51	13.78	Metropolitan Toronto
M1W	30	49000	8.74	5.9	12.48	Metropolitan Toronto
M3C	30	39000	11.07	7.47	15.81	Metropolitan Toronto
M6H	30	44000	9.76	6.59	13.94	Metropolitan Toronto
M8V	30	35000	12.26	8.28	17.51	Metropolitan Toronto
M9W	30	41000	10.47	7.07	14.95	Metropolitan Toronto
S0L	30	31000	13.86	9.35	19.79	Saskatchewan
S4N	30	30000	14.14	9.54	20.18	Saskatchewan
S6V	30	41000	10.45	7.05	14.92	Saskatchewan
S7M	30	31000	13.91	9.38	19.86	Saskatchewan
T5E	30	31000	13.62	9.19	19.44	Alberta
T5Y	30	41000	10.39	7.01	14.83	Alberta
T6H	30	31000	13.97	9.43	19.95	Alberta
T6W	30	36000	11.76	7.93	16.79	Alberta
V3E	30	37000	11.69	7.88	16.68	British Columbia
V3R	30	57000	7.58	5.12	10.82	British Columbia
V3T	30	36000	11.91	8.04	17	British Columbia
V5N	30	34000	12.57	8.48	17.94	British Columbia
A0K	25	27000	13.25	8.58	19.57	Newfoundland and Labrador
L4H	25	63000	5.65	3.66	8.34	Central Ontario
L6R	25	84000	4.26	2.76	6.29	Central Ontario
L8H	25	26000	13.7	8.86	20.22	Central Ontario
L8L	25	32000	11.32	7.33	16.71	Central Ontario
M1J	25	36000	9.81	6.35	14.48	Metropolitan Toronto
M2M	25	33000	10.99	7.11	16.22	Metropolitan Toronto

FSA	Cases	Population, Rounded	Incidence per 100,000 individuals per year, Rounded	Lower CI (95%), Rounded	Upper CI (95%), Rounded	Province
M3A	25	35000	10.35	6.7	15.27	Metropolitan Toronto
M5V	25	40000	8.94	5.79	13.2	Metropolitan Toronto
M6B	25	29000	12.36	8	18.24	Metropolitan Toronto
M6E	25	38000	9.4	6.09	13.88	Metropolitan Toronto
M6J	25	31000	11.59	7.5	17.11	Metropolitan Toronto
M6P	25	39000	9.16	5.93	13.52	Metropolitan Toronto
M9V	25	56000	6.38	4.13	9.42	Metropolitan Toronto
N3C	25	27000	13.44	8.7	19.83	Southwestern Ontario
S4X	25	26000	13.83	8.95	20.41	Saskatchewan
S7L	25	39000	9.14	5.91	13.49	Saskatchewan
S7N	25	28000	12.82	8.3	18.92	Saskatchewan
T0G	25	43000	8.29	5.36	12.23	Alberta
T1H	25	26000	13.6	8.8	20.07	Alberta
T4P	25	30000	11.89	7.7	17.56	Alberta
T6L	25	52000	6.88	4.45	10.15	Alberta
T9E	25	29000	12.15	7.87	17.94	Alberta
V3N	25	38000	9.33	6.04	13.77	British Columbia
V5C	25	29000	12.15	7.86	17.93	British Columbia
V5H	25	37000	9.56	6.18	14.11	British Columbia
V6P	25	31000	11.6	7.51	17.13	British Columbia
V7A	25	26000	13.49	8.73	19.91	British Columbia
L4B	20	36000	8.02	4.9	12.38	Central Ontario
L4T	20	39000	7.37	4.5	11.38	Central Ontario
L5C	20	30000	9.48	5.79	14.65	Central Ontario
L5V	20	51000	5.62	3.44	8.69	Central Ontario
L6V	20	42000	6.8	4.15	10.5	Central Ontario
L6W	20	22000	12.89	7.88	19.91	Central Ontario
L6X	20	61000	4.71	2.88	7.28	Central Ontario
L7A	20	73000	3.93	2.4	6.08	Central Ontario
M1L	20	34000	8.39	5.13	12.97	Metropolitan Toronto
M1R	20	29000	9.72	5.94	15.01	Metropolitan Toronto
M1T	20	34000	8.29	5.06	12.8	Metropolitan Toronto
M3N	20	42000	6.75	4.12	10.42	Metropolitan Toronto
M4M	20	24000	11.94	7.3	18.45	Metropolitan Toronto
M6C	20	24000	11.69	7.14	18.06	Metropolitan Toronto
M6K	20	38000	7.49	4.58	11.57	Metropolitan Toronto
M6N	20	41000	6.92	4.23	10.69	Metropolitan Toronto
R2P	20	30000	9.45	5.77	14.6	Manitoba
R3G	20	25000	11.36	6.94	17.55	Manitoba
S0M	20	48000	5.96	3.64	9.21	Saskatchewan
T3J	20	72000	3.99	2.44	6.17	Alberta
T5A	20	34000	8.3	5.07	12.81	Alberta
T6E	20	22000	12.75	7.79	19.69	Alberta

FSA	Cases	Population, Rounded	Incidence per 100,000 individuals per year, Rounded	Lower CI (95%), Rounded	Upper CI (95%), Rounded	Province
T8H	20	26000	11.06	6.76	17.09	Alberta
T8L	20	24000	11.99	7.32	18.51	Alberta
T8V	20	34000	8.45	5.16	13.04	Alberta
V0C	20	28000	10.16	6.2	15.69	British Columbia
V2N	20	32000	9.06	5.53	13.99	British Columbia
V3X	20	25000	11.35	6.93	17.53	British Columbia
V5A	20	24000	11.97	7.31	18.49	British Columbia
V5B	20	22000	12.76	7.79	19.71	British Columbia
V5E	20	26000	10.88	6.65	16.8	British Columbia
V5K	20	23000	12.55	7.66	19.38	British Columbia
V5S	20	28000	10.11	6.18	15.62	British Columbia
V6B	20	27000	10.55	6.44	16.29	British Columbia
V6Y	20	46000	6.18	3.77	9.54	British Columbia
V7C	20	39000	7.29	4.45	11.25	British Columbia
Y1A	20	27000	10.73	6.55	16.56	Yukon
